# Pulmonary regional blood flow: validation of low-dose two-volume dynamic CT perfusion imaging in a swine model

**DOI:** 10.1186/s41747-025-00556-3

**Published:** 2025-02-18

**Authors:** Yixiao Zhao, Nile Luu, Logan Hubbard, Shant Malkasian, Sabee Molloi

**Affiliations:** Department of Radiological Sciences, University of California, Irvine, Irvine, CA USA

**Keywords:** Computed tomography angiography, Lung, Perfusion imaging, Radiation dosage, Swine

## Abstract

**Background:**

We aimed to validate a low-dose two-volume pulmonary computed tomography (CT) perfusion technique.

**Methods:**

Five Yorkshire swine (weight 53.6 ± 2.6 kg) underwent 21 independent CT perfusion acquisitions. Intravenous contrast material (370 mg/mL iodine, 0.5 mL/kg) and saline chaser (0.5 mL/kg) were injected at 5 mL/s for each acquisition. Two-volume and multivolume dynamic CT perfusion data were acquired using a 320-slice CT, with multivolume measurements serving as the reference standard. The two-volume CT perfusion involved a low-dose (50 mA) volume scan before contrast injection and a diagnostic (300 mA) volume scan after bolus-tracking in the main pulmonary artery at the peak contrast enhancement. Multivolume CT perfusion included 15–20 volume scans for blood flow measurement. Paired sample *t*-test, linear regression, and Bland–Altman analysis compared both global and regional two-volume perfusion measurements to the reference standard. The reproducibility of the two-volume CT perfusion was assessed from two independent measurements under the same perfusion condition.

**Results:**

Two-volume global perfusion measurements (*P*_2V_) were related to reference multivolume (*P*_MV_) measurements by *P*_2V_ = 0.96 × *P*_MV_ + 0.45 (*r* = 0.92), with a root-mean-square error of 1.29 mL/min/g and a root-mean-square deviation of 1.29 mL/min/g. The CT dose index for the two-volume and multivolume CT perfusion measurements were 9.3 mGy and 184.8 mGy, respectively.

**Conclusion:**

We successfully validated a prospective, two-volume CT perfusion technique in a swine model. The findings affirm the feasibility of accurate and reproducible pulmonary blood flow measurement.

**Relevance statement:**

This two-volume CT pulmonary perfusion technique, validated in a swine model, demonstrates the feasibility of blood flow measurement with a substantial reduction in radiation exposure. It could allow low-dose regional blood flow measurement in the assessment of pulmonary artery disease in humans.

**Key Points:**

- Lung perfusion can be measured in mL/min/g using a prospective, two-volume CT technique.
- Flow measurement is achievable in a swine model with a radiation dose as low as 9.3 mGy.
- CT angiography and perfusion can be acquired following a single contrast injection.

**Graphical Abstract:**

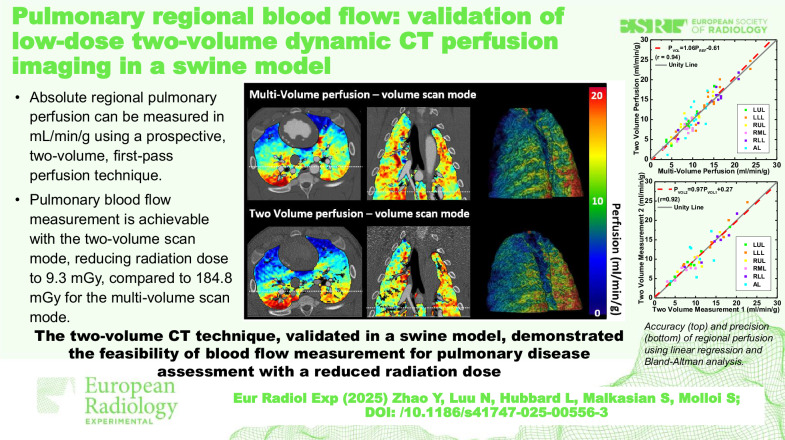

## Background

Pulmonary computed tomography (CT) angiography is considered the standard of care for diagnosing pulmonary embolism (PE), offering high specificity and sensitivity [1, 2]. While dual-energy pulmonary CT angiography provides valuable insights into relative perfusion defects, it lacks the ability to offer quantitative lung perfusion measurements in mL/min/g [3–5]. Additionally, current dual-energy CT techniques are limited in their ability to quantify pulmonary microvascular disease, which is critical in conditions such as diabetes [6], chronic obstructive pulmonary disease (COPD), and emphysema [7], all of which require more accurate pulmonary blood flow measurement.

A quantitative approach to pulmonary perfusion holds significance for diagnosing and assessing the severity of pulmonary artery disease over time and across patient groups, including PE and chronic thromboembolic hypertension, revealing information not available from a standard pulmonary CT angiogram and relative perfusion assessment. Pulmonary perfusion can differentiate diseases caused by obstruction of the large pulmonary arteries from those resulting from diffuse and small vessel disease, as these conditions have different prognoses and require different treatments. Current dynamic CT perfusion methods can provide additional information in quantitatively assessing different pulmonary diseases and lung cancer [8–12]. However, these methods involve a high radiation dose and are limited by their reproducibility and accuracy. Existing techniques utilize several small volumes of interest in the lung tissue as the perfusion compartment for blood flow measurement, but they underestimate blood flow because contrast exits the compartment during the time interval when images are acquired for flow measurement [10, 13]. Additionally, dynamic CT perfusion techniques such as the maximum slope model and the deconvolution model require 15–20 volume scans for a complete contrast pass curve, leading to a high radiation dose and motion misregistration artifacts [12–15]. These limitations have hampered the routine clinical application of dynamic CT perfusion.

We have recently reported a dynamic CT perfusion technique based on a large perfusion compartment and a multivolume scan mode but using only two volume scans for flow measurement [16]. The accuracy of this technique was initially validated using fluorescent microspheres by acquiring 15–20 volume scans but systematically selecting only two volume scans for blood flow measurement [16]. The next step is to validate a low-dose, prospectively acquired two-volume dynamic CT perfusion technique.

Therefore, the purpose of this study was to validate a prospective, low-dose, CT perfusion technique by acquiring only two-volume scans for global and lobar lung perfusion measurement. The central hypothesis was that both global and regional perfusion can be measured using a two-volume perfusion technique, as compared with a previously validated multivolume perfusion technique as a reference standard [16]. The two-volume perfusion technique is regarded as a lower radiation dose approach, as it involves prospectively acquiring only two-volume scans. In contrast, the previously reported multivolume perfusion technique is considered a higher radiation dose approach, as it requires acquiring 15–20 volume scans, yet only two volume scans are utilized for flow measurements [16].

## Methods

### General methods

**Fig. 1:**
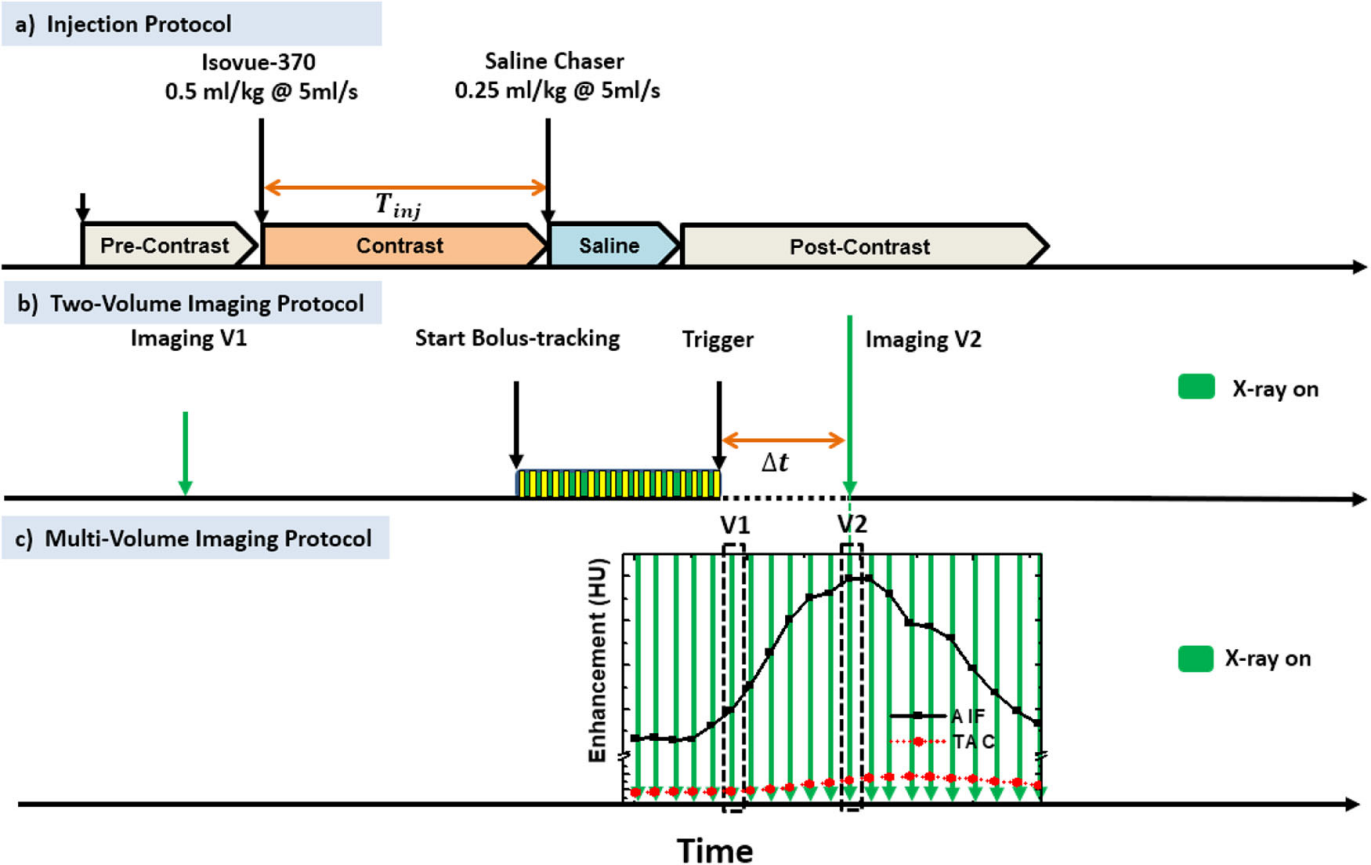
The timeline of the contrast injection, multi-volume, and two-volume CT acquisition protocol. **a** Injection protocol. Contrast-enhanced imaging, contrast was injected at 5 mL/s followed by a saline chaser at the same rate. Dynamic imaging of the lung was then performed. **b** Two-volume CT imaging protocol. The first volume scan (V1) was acquired before contrast injection. The second volume scan (V2) was acquired at the maximum enhancement of AIF using the bolus-tracking technique and a pre-defined time-to-peak delay. ${T}_{{inj}}$, Contrast injection time; $\varDelta t$, Time-to-peak delay. **c** Multi-volume CT imaging protocol. The entire pulmonary arterial input function (AIF) and lung tissue time attenuation curve (TAC) were captured and used for perfusion measurement

All animal procedures adhered to the guidelines for animal care and received approval from the Animal Care Committee (see “Declarations”). The validation of the two-volume dynamic CT perfusion technique involved five male Yorkshire swine weighing 53.6 ± 2.6 kg (mean ± standard deviation). For each swine, 2–4 two-volume dynamic CT perfusion scans were acquired. The two-volume CT perfusion scans were performed by initially acquiring the first non-contrast volume scan (V1), followed by contrast injection and acquisition of the second volume scan after a bolus tracking trigger and a predefined time-to-peak delay (V2) (Fig. 1) [17]. The two-volume scans were repeated after approximately 10 min to assess the reproducibility of perfusion measurement. Multivolume dynamic CT perfusion consisting of 15–20 volume scans, serving as the reference standard, was acquired within approximately 15 min after the two-volume scans. The multivolume technique was previously validated using fluorescent microspheres [16] and was used to assess the accuracy of the two-volume technique. In two animals, an additional pair of two-volume scans, followed by a multivolume scan, were acquired within approximately 2–5 h of the first set of scans to assess the technique in a lower perfusion range as perfusion gradually reduces over time in an anesthetized animal.

The study encompassed 21 successful acquisitions for global and regional perfusion analyses, comprising 14 two-volume scans and their associated 7 reference multivolume scans. In the accuracy assessment, three animals underwent one multivolume scan paired with two two-volume acquisitions, while the remaining two animals each had two multivolume scans and four two-volume scans. For precision assessment, seven pairs of two-volume scans were utilized, with reproducibility exclusively compared within each pair. Six three-dimensional lobar territories per animal were considered for regional perfusion analysis, resulting in a total of 126 (21 × 6) regional segments evaluated. The experimental data were successfully acquired and analyzed between November 2020 and June 2021, with all authors participating in data acquisition, and authors Y.Z. and N.L. conducting data analyses.

### Animal preparation

Each swine was initially induced with Telazol (4.4 mg/kg) and Xylazine (2.2 mg/kg). Anesthesia was maintained through mechanical ventilation with 1.5–2.5% Isoflurane (Highland Medical Equipment, Temecula, CA, and Baxter, Deerfield, IL, USA) after intubation. Two introducer sheaths (AVANTI®, Cordis Corporation, Miami Lakes, FL, USA) were placed in the left femoral and left jugular veins for intravenous fluid and contrast material administration. An additional sheath was inserted into the right femoral artery for invasive pressure monitoring. A urinary catheter was placed in the bladder for urine drainage. Vital signs, including heart rate, end-tidal carbon dioxide, oxygen saturation, and mean arterial pressure, were recorded every 15 min. At the experiment’s conclusion, animals were euthanized with saturated KCl under deep anesthesia.

### Scanning protocol

All CT examinations utilized a 320-row detector CT scanner (Aquilion One, Canon Medical Systems, Tustin, CA, USA). Animals were positioned in a supine position for all scans. The tube voltage was set at 100 kV, with a scan field-of-view of 320 mm and a gantry rotation time of 0.35 s. For two-volume acquisition, a low-dose non-contrast volume scan (V1) was acquired with breath-hold, followed by intravenous contrast injection (0.5 mL/kg Isovue-370, Bracco Diagnostics, Monroe Township, NJ, USA) and saline chaser (0.25 mL/kg) at 5 mL/s using an automatic injector (Empower CTA, Acist Medical Systems, Eden Prairie, MN, USA) (Fig. 1). The second volume scan (V2) was acquired following a bolus tracking trigger within the pulmonary artery trunk and a delay time based on the injection time interval (Fig. 1) [17]. The multivolume acquisition involved dynamic CT scans at end-inspiration for 15–20 cardiac cycles under ECG gating following intravenous contrast injection. A minimum 10-min delay was employed between acquisitions for contrast clearance. Two-volume scans used a tube current of 50 mA for V1 and 300 mA for V2, which also served as the CT angiogram. Multivolume scans were acquired with a constant tube current of 300 mA. A collimation of 320 × 0.5 mm was used for volume scans. Full projection data, lung FC07 kernel, and an adaptive iterative dose reduction three-dimensional reconstruction algorithm were employed.

### Perfusion measurement

**Fig. 2:**
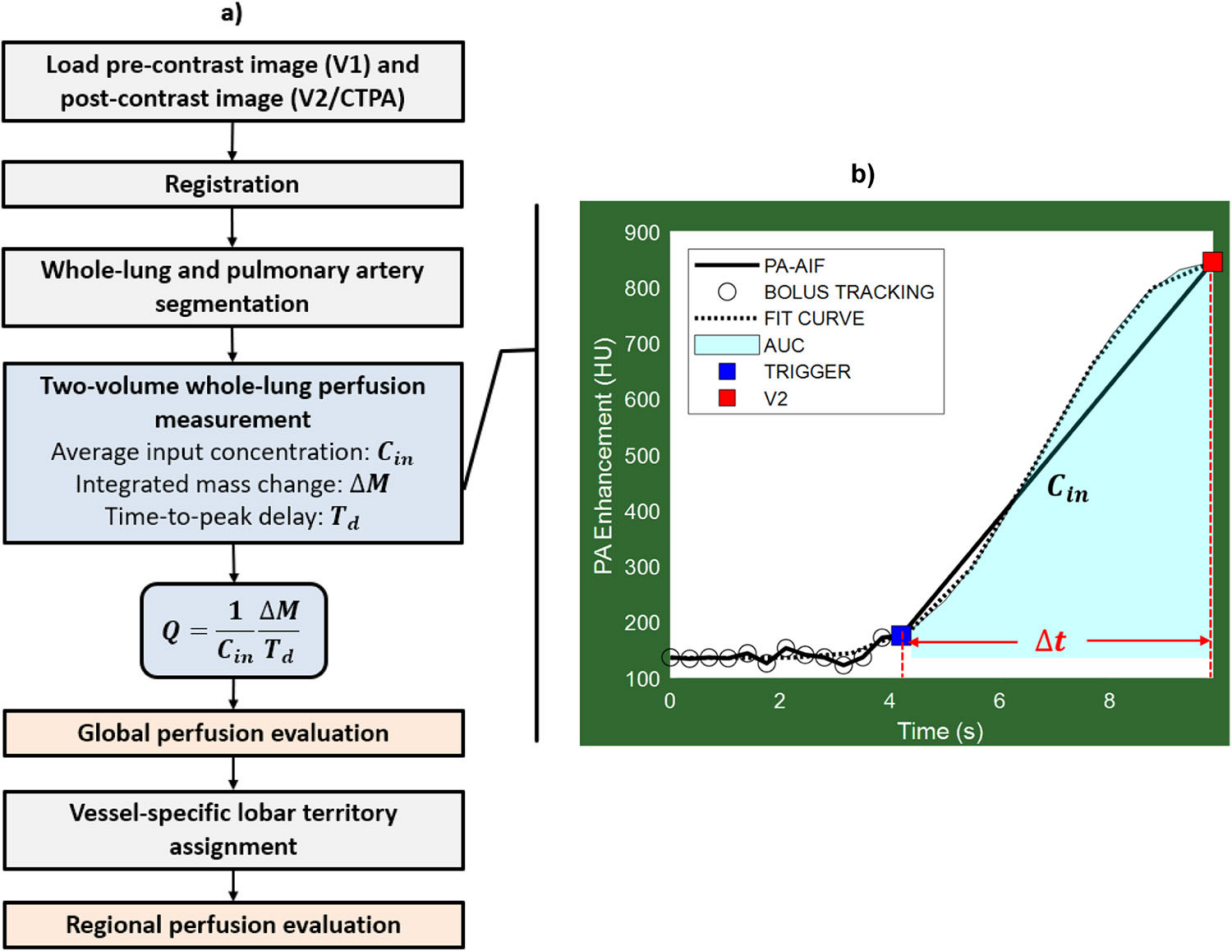
Image processing flowchart and two-volume perfusion technique (**a**). The first volume scan (V1) was acquired before contrast injection. The second volume scan (V2) was acquired after the bolus-tracking technique and a pre-defined time-to-peak delay (**b**). $\varDelta M$, Integrated contrast mass change; ${C}_{{in}}$, Average input concentration; $\varDelta t$, Time-to-peak delay; BT, Bolus-tracking; AIF, Arterial input function; AUC, Area under the curve

The average pulmonary blood flow was determined using a first-pass, whole-lung compartment model, as previously reported [14]. Preceding the contrast outflow from the entire compartment, the average pulmonary perfusion (*P*, mL/min/g) correlates with the integrated contrast mass change within the compartment (${{{\Delta}}{{M}}}_{{{{\mathrm{c}}}}}$, mg). It is normalized by the average arterial input concentration (*C*_in_, mg/mL), lung tissue mass (${{M}}_{{{{\mathrm{T}}}}}$, g), and the total contrast accumulation time ($\Delta {t},{{{\mathrm{min}}}}$), as illustrated in Fig. 2. Specifically, ∆*t* represents the time-to-peak delay between the base and the peak of arterial enhancement, predetermined based on a patient-specific injection time interval technique [17].

1 $${P}=\frac{1}{{{{M}}}_{{{{\mathrm{T}}}}}}\frac{{{1}}}{{{{{C}}}}_{{{{\mathrm{in}}}}}}\,\frac{{{{\Delta}}{{M}}}_{{{c}}}}{\Delta {{t}}}$$

The absolute perfusion mL/min/g was further normalized on a voxel-by-voxel basis, accounting for the tissue mass’s heterogeneity, which considers the tissue-air fraction [16]. Conveniently, for CT imaging, the contrast enhancement in HU is linearly related to the contrast concentration (in mg/mL), where the imaging kVp determines a constant slope of relation. Hence, the contrast enhancement within the blood and tissue directly equals the contrast concentration within the blood and tissue multiplied by the same constant slope of relation. Therefore, in Eq. 1, the units for compartmental contrast mass accumulation rate ($\frac{{{{\Delta }}{{M}}}_{{{{\mathrm{c}}}}}}{\Delta{{t}}}$) are HU/min, and units for the average incoming contrast concentration (*C*_in_) are HU/mL, so HU cancels out in the ratio (see Eq. 1).

For the two-volume perfusion measurement, V1 was acquired before contrast injection with a breath-hold at maximal end-inspiration, and V2 was acquired following contrast injection at the maximal arterial enhancement with another breath-hold at full inspiration (Fig. 2b). Specifically, bolus-tracking (SureStart, Aquilion One, Canon Medical Systems, Tustin, CA, USA) was performed using a 2-mm image window at the pulmonary artery level, triggered after the region-of-interest enhancement exceeded a threshold of 80 HU above the baseline blood pool enhancement. Additionally, a time delay was set to ensure V2 was captured near the arterial enhancement peak for an adequate contrast-to-noise ratio. The time-to-peak delay (∆t) can be predetermined by relating it to one-half of the contrast injection time (${T}_{{inj}}$) plus a dispersion delay to the pulmonary artery [17].

In the multivolume measurement, the two-volume scans (V1 and V2) were deliberately chosen by one operator (Y.Z., with 4 years of cardiovascular CT image processing experience) from the contrast pass curve, aligning with approximately the base and peak of the pulmonary arterial enhancement, as illustrated in Fig. 1b. The accuracy of the reference multivolume perfusion measurement was previously affirmed using fluorescent microsphere measurements as the established reference standard [16].

### Image processing

The image processing procedure is depicted in Fig. 2. The non-contrast and contrast volume scans underwent registration using a deformable affine-based algorithm [18] using available software (https://github.com/KCL-BMEIS/niftyreg; NiftyReg; London, United Kingdom). The higher-dose image with contrast opacification was used as the “fixed” image in the registration process with the non-contrast image. The contrast-enhanced image was then utilized to generate the flow map. Two operators (Y.Z. and N.L., with 4 years and 2 years of cardiovascular CT image processing experience, respectively) segmented an arterial input function by employing a cylindrical volume-of-interest in the pulmonary artery, while the entire lung parenchymal volume-of-interest served as the perfusion compartment. Semiautomatic segmentation of these two volume-of-interests was carried out using a Vitrea workstation (Vitrea fX version 7.7, Vital Images, Inc., Minnetonka, MN, USA). Subsequently, the integrated contrast mass change of the entire lung parenchyma and the average input concentration were computed, yielding a global lung perfusion measurement through in-house software (MATLAB 2021a, MathWorks, Natick, MA, USA). The entire lung was then segmented into six lobar segments, employing an automated vessel-specific lobar assignment based on a minimum-cost path technique [19]. These segments include the left upper, left lower, right upper, right middle, right lower, and accessory lobes. Mean perfusion values for each lobe were calculated to facilitate regional lung perfusion comparisons.

### Statistical analysis

The study assessed the accuracy and precision of the two-volume perfusion technique in measuring both global and regional perfusion. Perfusion values were presented as mean ± standard deviation. To evaluate the accuracy, two-volume perfusion measurements were compared to reference multivolume measurements using paired-sample *t*-test, linear regression, and Bland–Altman analysis. For precision assessment, two independent perfusion measurements were compared through paired-sample *t*-test, linear regression, and Bland–Altman analysis. Statistical significance was determined with *p*-values less than 0.05. Additionally, 95% confidence intervals for fitting parameters were provided. The study also calculated root-mean-square-error (RMSE), root-mean-square deviation (RMSD), Pearson correlation coefficient (*r*), and Lin’s concordance correlation coefficient [20] were also computed. The Shapiro–Wilk test [21] and kernel density estimate plots were employed using Python (https://docs.scipy.org/doc/scipy/reference/generated/scipy.stats.shapiro.html) to assess the normality of perfusion measurements. Based on both statistical analysis and visualization, perfusion measurements from both the prospective and retrospective protocols demonstrated normal distributions. All statistical analyses were performed using SPSS software (version 22, IBM Corporation, Armonk, NY, USA).

## Results

### Vital parameters

The mean (±standard deviation) heart rate and arterial pressure across all CT perfusion acquisitions were 88.5 ± 19.3 bpm and 58.5 ± 17.7 mmHg, respectively, with cardiac output ranging from 1.9 L/min to 5.2 L/min.

### Qualitative analysis

**Fig. 3:**
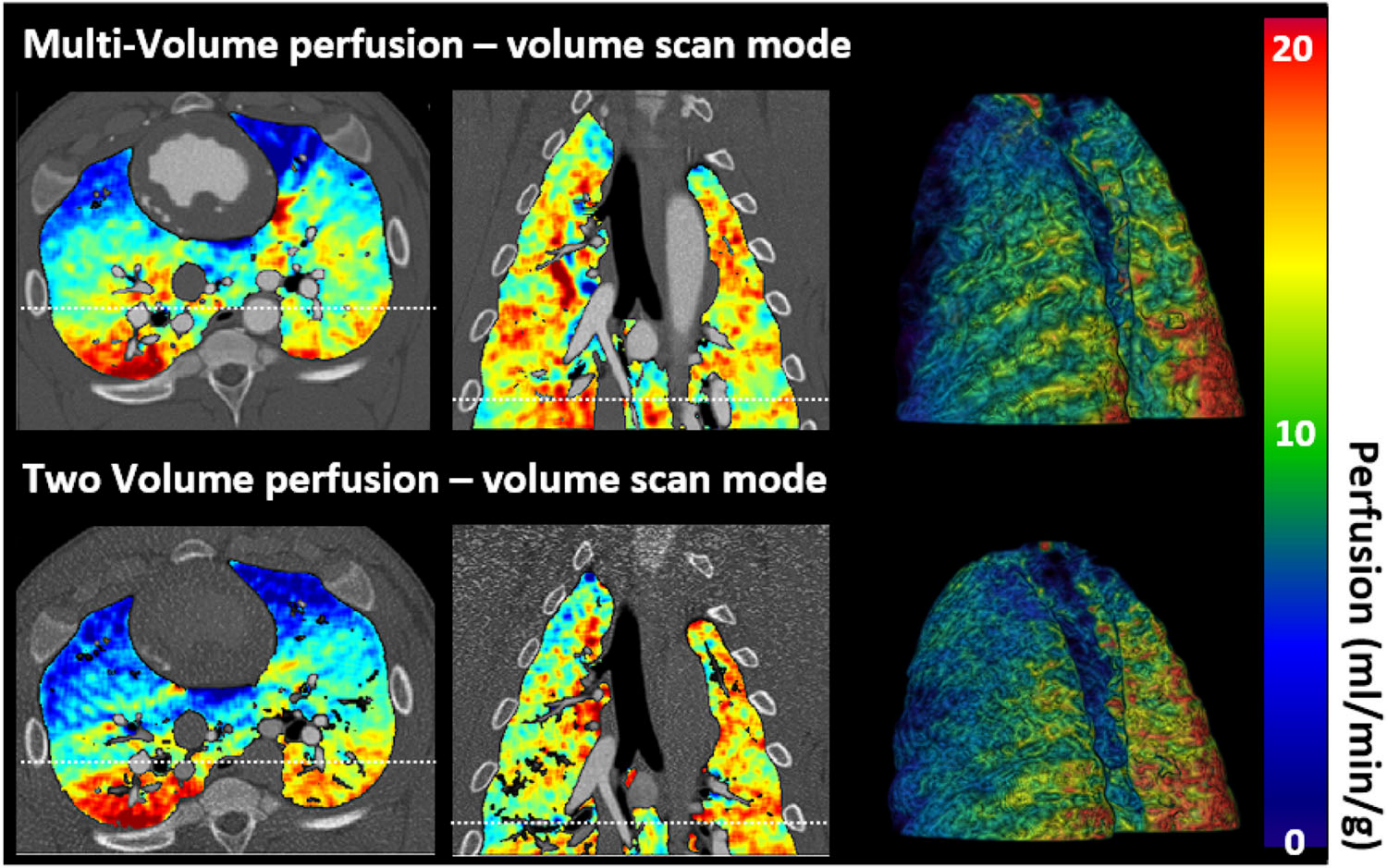
Qualitative perfusion maps with two-volume and multivolume perfusion techniques. Top, reference multivolume perfusion map. Bottom, two-volume perfusion map. The bar on the right shows the absolute perfusion measurement in mL/min/g

Figure 3 illustrates perfusion maps obtained using two-volume and multivolume CT perfusion techniques in axial, coronal, and three-dimensional volumetric images. Perfusion measurements, presented in mL/min/g, use red color for high and blue color for low perfusion values.

### Quantitative analysis of accuracy

**Fig. 4:**
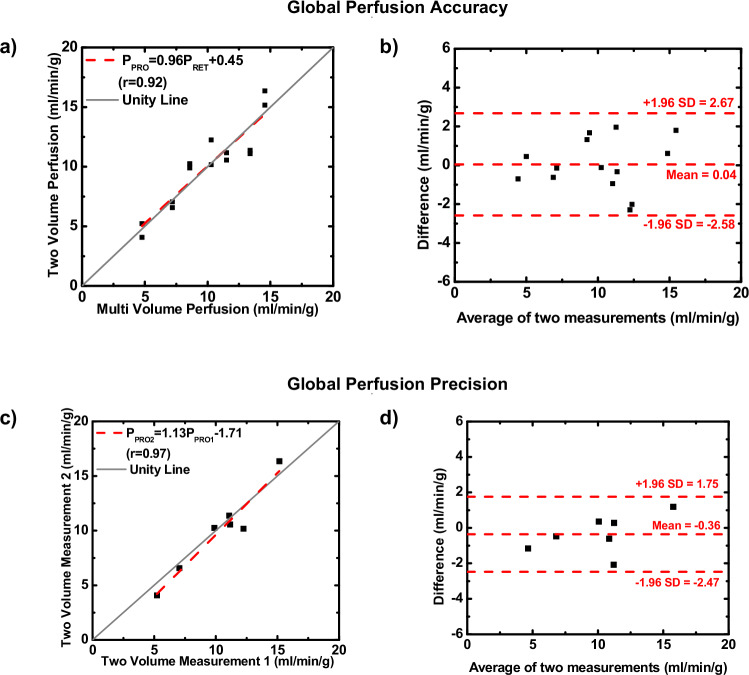
Accuracy and precision of global perfusion using linear regression and Bland–Altman analysis. **a**, **b** Accuracy assessments between the two-volume measurement and reference multivolume measurement. **c**, **d** Precision assessments between two independent two-volume measurements

Average global perfusion for two-volume and reference multivolume measurements was 10.09 ± 3.46 mL/min/g and 10.04 ± 3.46 mL/min/g, respectively (*p* = 0.902) (Table 1). Linear regression indicated that the two-volume measurements (*P*_2V_) were related to the reference multivolume measurements (*P*_MV_) by *P*_2V_ = 0.96 × *P*_MV_ + 0.45 (*r* = 0.92), with an RMSE of 1.29 mL/min/g and a RMSD of 1.29 mL/min/g (Fig. 4a and Table 1). The mean difference of the global perfusion measurements as compared with reference multivolume measurements was 0.04 mL/min/g, where all measurements were within limits of agreement, as shown in Fig. 4b.

**Table 1:** Evaluation of the accuracy of the perfusion measurement: two-volume measurement *versus* the reference multivolume measurement

Lung segments	Number of pairs	Multivolume CT perfusion, (mL/min/g)	Two-volume CT perfusion, (mL/min/g)	*p*-value (α < 0.05)	RMSE, (mL/min/g)
Global perfusion	14	10.04 ± 3.46	10.09 ± 3.46	0.902	1.29
Right lung					
Right upper lobe	14	9.70 ± 3.29	10.06 ± 3.43	0.387	1.48
Right middle lobe	14	7.81 ± 2.45	7.11 ± 2.51	0.063	1.42
Right caudal lobe	14	14.54 ± 4.53	14.35 ± 4.67	0.569	1.42
Accessory lobe	14	9.94 ± 3.19	10.04 ± 4.35	0.891	2.46
Left lung					
Left upper lobe	14	8.78 ± 3.77	9.27 ± 4.71	0.230	1.46
Left caudal lobe	14	14.06 ± 5.43	14.37 ± 5.92	0.578	1.96

The mean perfusion values are the average perfusion values of each lobe sample, the standard deviation values are the perfusion value variations from the same sample, not the measurement errors. The *p*-values were calculated by paired-sample *t*-test

*RMSE* Root-mean-square error

**Fig. 5:**
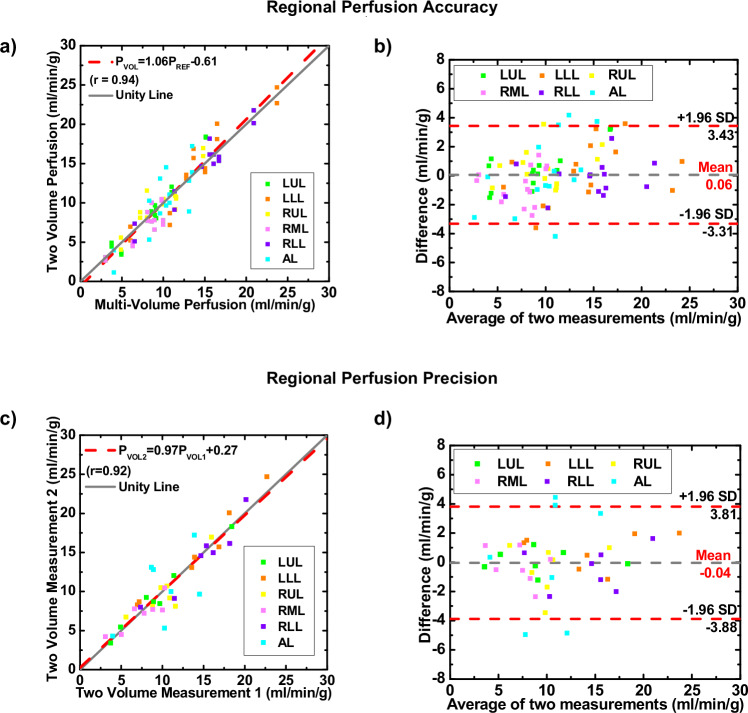
Accuracy and precision of regional perfusion using linear regression and Bland–Altman analysis. **a**, **b** Accuracy assessment. **c**, **d** Precision assessment. AL, Accessory lobe; LLL, Left lower lobe; LUL, Left upper lobe; RLL, Right lower lobe; RML, Right middle lobe; RUL, Right upper lobe

Regional perfusion showed no significant differences between the two-volume measurements and the reference multivolume measurements (*p* ≥ 0.756. Linear regression indicated that the overall two-volume regional perfusion measurements were related to the reference multivolume measurements by *P*_2V_ = 1.06 *P*_MV_ − 0.61 (*r* = 0.94), with a RMSD of 1.69 mL/min/g and a RMSE of 1.71 mL/min/g (Fig. 5a, b and Table 1). The linear regression parameters of overall regional perfusion measurements are shown in Table 2. Confidence intervals were wider for regional perfusion compared to global perfusion (Figs. 4b and 5b), with relatively small systemic bias (mean difference = 0.06 mL/min/g).

**Table 2:** Evaluation of linear regression parameters for accuracy (two-volume *versus* reference multivolume perfusion measurement) and reproducibility (two-volume 1 *versus* two-volume 2 perfusion measurement)

	Slope	Intercept	Pearson *r*	CCC	RMSE, (mL/min/g)	RMSD, (mL/min/g)
Accuracy						
Global (*n* = 14)	0.96 (0.71, 1.21)	0.45 (-2.21, 3.11)	0.92 (0.79, 0.97)	0.92 (0.79, 0.97)	1.29	1.29
Regional overall (*n* = 84)	1.06 (0.98, 1.15)	-0.61 (-1.59, 0.36)	0.94 (0.84, 0.98)	0.93 (0.83, 0.98)	1.71	1.69
Precision						
Global (*n* = 7)	1.13 (0.79, 1.48)	-1.71 (-5.40, 1.97)	0.97 (0.86, 0.99)	0.95 (0.80, 0.99)	1.06	1.67
Regional overall (*n* = 42)	0.97 (0.84, 1.11)	0.27 (-1.31, 1.85)	0.92 (0.69, 0.98)	0.92 (0.69, 0.98)	1.92	1.92

Data in parentheses are 95% confidence intervals

*CCC* Concordance correlation coefficient, *RMSD* Root-mean-square deviation

### Quantitative analysis of reproducibility

Reproducibility of two-volume global perfusion measurements revealed no significant difference between the two paired sets with average perfusions of 10.26 ± 3.30 and 9.91 ± 3.86 mL/min/g (*p* = 0.414). Linear regression indicated that the first (*P*_V1_) and second (*P*_V2_) two-volume regional perfusion measurements were related by *P*_V2_ = 1.13 × *P*_V1_ − 1.71 (*r* = 0.97), with an RMSD of 1.67 mL/min/g and a RMSE of 1.06 mL/min/g). Regression and Bland–Altman analyses of the global two-volume measurements are shown in Fig. 4c, d and detailed in Tables 2 and 3.

**Table 3:** Evaluation of reproducibility of the perfusion measurement: two-volume measurement 1 *versus* two-volume measurement 2

Lung segments	Number of pairs	Two-volume CT perfusion 1, (mL/min/g)	Two-volume CT perfusion 2, (mL/min/g)	*p*-value (α < 0.05)	RMSE
Global perfusion	7	10.26 ± 3.30	9.91 ± 3.86	0.414	1.06
Right lung					
Right upper lobe	7	10.45 ± 3.12	10.05 ± 3.36	0.545	1.61
Right middle lobe	7	7.37 ± 2.65	7.08 ± 2.15	0.569	1.20
Right caudal lobe	7	14.76 ± 4.26	14.35 ± 4.63	0.494	1.43
Accessory lobe	7	10.18 ± 3.56	10.35 ± 4.54	0.913	3.70
Left lung					
Left upper lobe	7	9.30 ± 4.82	9.38 ± 4.82	0.807	0.74
Left caudal lobe	7	14.19 ± 5.73	14.99 ± 5.90	0.135	1.40

The mean perfusion values are the average perfusion values of each lobe sample, the standard deviation values are the perfusion value variations from the same sample, not the measurement errors. The *p*-values were calculated by paired-sample *t*-test

*RMSE* Root-mean-square error

Regional perfusion reproducibility showed that the first and second two-volume regional perfusion measurements were related by *P*_V2_ = 0.97 *P*_V1_ + 0.27 (*r* = 0.92), with a RMSD of 1.92 mL/min/g and a RMSE of 1.92 mL/min/g (Fig. 5c, d and Table 3). Nonsignificant differences between the two two-volume measurements were observed for all lobes (*p* ≥ 0.971). The accessory lobe had a noticeably higher RMSE than all other lobar segments for two-volume measurements (Table 3). Overall, good correlations were found between the two two-volume measurements with less bias and variations as compared with the reference multivolume measurements (Fig. 5c, d and Table 3).

### Radiation dose estimation

The average CT dose index for two-volume and multivolume scans was 9.3 mGy and 184.8 mGy, respectively. The calculated effective dose for two-volume and multivolume scans were 2.7 mSv and 53.8 mSv, respectively. Detailed dose estimations and scan parameters are provided in Table 4.

**Table 4:** Scan parameters and x-ray dose estimation of the multivolume and two-volume perfusion measurements

	Multivolume	Two-volume		
	Volume scans (*n* = 20)	V1 (*n* = 1)	V2 (*n* = 1)	V1 + V2
Scan parameters				
Tube current (mA)	300	90	300	N/A
Tube voltage (kV)	100			
Detector collimation (mm)	320 × 0.5	320 × 0.5		
Dimension measurements				
Anterior-posterior (cm)	24.2 ± 1.0			
Lateral (cm)	30.8 ± 1.3			
Effective diameter (cm)	27.3 ± 0.8			
*z*-length (cm)	16			
X-ray dose estimation				
CT dose index (mGy)	184.8	2.1 ± 0.2	7.2 ± 0.2	9.3 ± 0.4
Size-specific dose estimate (mGy)	240.0	2.71	9.4	12.01
Dose length product (mGy × cm)	3,843.7	192.9		
Effective dose (mSv)	53.8	2.7		

Effective diameter $=\,\sqrt{{{\rm{AP}}}\times{{\rm{LAT}}}}$. Size-specific dose estimate $={{\rm{CT}}}\;{{\rm{dose}}}\;{{\rm{index}}\,}_{{\rm{VOL}}}^{32} \, \times \, {f}_{{\rm{size}}}^{32}$ where $f_{{\rm{size}}}^{32}$ is a value derived from the measured effective diameter for a 32-cm phantom. In this case, the $f_{{\rm{size}}}^{32}$ was equal to 1.3. Effective dose = Dose length × *k*, where *k* is a coefficient derived for an adult. In this case, *k* = 0.014

## Discussion

The two-volume dynamic CT lung perfusion technique was validated using multivolume perfusion measurements as the reference standard. The results show that both global and regional perfusion measurements were in good agreement with the previously validated multivolume measurements reference [16]. For regional perfusion measurements, near unity, regression slope, and negligible bias were measured. Additionally, the results show excellent reproducibility of the two-volume perfusion technique, with good correlation, negligible bias, and relatively small RMSE and RMSD. More importantly, the results of two-volume CT perfusion measurements indicate that accurate perfusion measurement is feasible with the two-volume dynamic CT perfusion technique. Lastly, the radiation dose for the two-volume perfusion measurement and the CT angiogram was very low, which ultimately enables the clinical application of the technique for the assessment of pulmonary artery diseases such as acute or chronic PE.

Existing multivolume CT perfusion techniques typically define small volumes of interest as the perfusion compartment. In contrast, the two-volume dynamic CT perfusion technique considers both lungs as a single lumped compartment, effectively merging the intravascular, interstitial, and cellular sub-compartments into one entity. This approach simplifies the model without compromising its validity, as the transfer of contrast mass between intravascular and extravascular compartments is not a limiting factor.

The substantial dynamic change in contrast mass within this large compartment during the measurement period allows accurate quantification of the total mass change using a pre-contrast scan (acquired before contrast injection) and a post-contrast scan (acquired near the peak of pulmonary arterial enhancement). By treating the left and right lungs as a single compartment, the measurement window—during which significant contrast mass outflow is minimal—is significantly extended. This window corresponds to the pulmonary transit time from the right to the left ventricle, approximately 6–8 s [22].

Thus, CT flow measurements derived during the first circulatory pass, or first-pass analysis, ensure that contrast mass outflow from the compartment is nearly zero or negligible relative to inflow. This is further supported by the pulmonary arterial time-to-peak enhancement, which typically occurs within 3–7 s, depending on the contrast injection protocol [17].

Previous studies have used dual-energy [3, 4], photon-counting [23] CT pulmonary angiography, as well as subtraction CT between unenhanced and enhanced CT scans [24-26] to show the iodine distribution map for the detection of suspected PE. As there is no temporal information, the iodine map can only reflect the relative iodine volume differences in the lung parenchyma in an unknown time point within the contrast pass curve, which lacks physiological information in terms of absolute blood flow or perfusion in mL/min/g. A first-pass kinetic model in conjunction with the temporal iodine concentration change estimation is required to provide perfusion measurement. Our two-volume CT perfusion technique minimized the number of volume scans required in a dynamic CT perfusion measurement to two scans and maximized the size of the perfusion compartment to the whole lung. The expansion of the compartment size enables accurate and robust perfusion measurement as the iodine accumulation is calculated by integrating the change of HU within the entire lung as a single compartment before calculating blood flow on a voxel-by-voxel basis [27]. Given that the contrast transit time within the entire lung is approximately five seconds, blood flow measurement can be completed before contrast starts to exit the compartment. Specifically, the starting time point is determined by bolus-tracking on the atrial input vessel as an indication of contrast delivery; while the end time point is determined when the arterial input vessel is at maximal enhancement. Such time-to-peak delay has been empirically related to the contrast injection duration and a fixed dispersion time in previous studies, demonstrating accurate and reliable time-to-peak prediction over a wide range of body weights and cardiac outputs [17]. More importantly, as the second volume scan is acquired at the maximum contrast enhancement, it can also be used as the optimal pulmonary CT angiogram [28]. Therefore, anatomical and physiological assessment of pulmonary disease is feasible with a single contrast injection at a low radiation dose.

Dual-energy pulmonary angiography produces an iodine map reflecting relative iodine volume differences in lung parenchyma or relative perfusion. In contrast, the two-volume dynamic CT perfusion technique provides absolute perfusion values in mL/min/g on a voxel-by-voxel basis. The critical consideration is whether absolute perfusion offers unique clinical insights not addressed by relative perfusion from dual-energy pulmonary angiography. Absolute perfusion allows quantification of disease severity across different lungs and patients, encompassing conditions affecting the entire lung. Moreover, it facilitates assessing the impact of interventions for PE, offering a comprehensive approach to evaluating disease severity and treatment outcomes.

Our results showed increased variance in regional perfusion measurement as compared with global perfusion measurements. There are a few potential contributors that may lead to an increase in regional measurement error. First, the lobar assignment technique is expected to generate slightly different three-dimensional volumes for repeated assignments, which is expected to introduce slight errors in repeated lobar perfusion measurements [19]. Second, regional blood flow measurements in a lobe are expected to have more measurement error as compared with global measurements since the measurements were made in smaller volumes. Finally, beam hardening artifacts due to contrast inflow can produce measurement error, particularly for the lobes adjacent to the heart and large vessels, resulting in an increased error in the accessory, right, and left middle lobes.

Other limitations are as follows. First, although the wide-volume scanner has a 16-cm *z*-coverage, it cannot scan the entire lung in one scan. As the proposed CT perfusion technique requires only two volumes, a helical scan mode may be necessary to further expand the scan window to the whole lung and allow for full analysis of the lung lobes. This is the focus of our current research. Second, a multivolume CT perfusion technique served as the reference standard in the quantitative assessment. Fortunately, the multivolume CT perfusion technique has previously been validated with fluorescent microsphere perfusion measurement as the reference standard [16]. Lastly, the study was conducted on a relatively small number of healthy swine. The two-volume perfusion technique needs to be further evaluated in humans with various cardiopulmonary conditions such as acute PE, pulmonary hypertension, as well as airway obstruction diseases.

To summarize, this study successfully validates a low-dose, two-volume CT perfusion technique in a swine model. The findings affirm the feasibility of accurate and reproducible blood flow measurements. This two-volume perfusion technique holds promise for enabling low-dose regional blood flow measurement in the assessment of pulmonary artery disease.

## Data Availability

Data generated or analyzed during the study are available from the corresponding author by request.

## Abbreviations

CT: Computed tomography
PE: Pulmonary embolism
RMSD: Root-mean-square deviation
RMSE: Root-mean-square error
